# Extramammary Paget’s diseases in men from the Shanghai area: its association with PSA level increase*

**DOI:** 10.1111/j.1600-0463.2010.02657.x

**Published:** 2010-10

**Authors:** GUOHAI SHI, DING-WEI YE, XUDONG YAO, SHILING ZHANG, BO DAI, HAILIANG ZHANG, YIJUN SHEN, YAO ZHU, YIPING ZHU, WENJUN XIAO, CHUNGUANG MA

**Affiliations:** 1 Department of Urology, Cancer Hospital, Fudan UniversityShanghai; 2 Department of Oncology, Shanghai Medical College, Fudan UniversityShanghai, China

**Keywords:** PSA, prostate cancer, EMPD disease

## Abstract

Shi G, Ye D-W, Yao X, Zhang S, Dai B, Zhang H, Shen Y, Zhu Y, Zhu Y, Xiao W, Ma C. Extramammary Paget’s diseases in men from the Shanghai area: its association with PSA level increase. APMIS 2010; 118: 777–81.

The aim of this study was to determine the incidence of prostate cancer in patients with extramammary Paget’s disease (EMPD). All cases of EMPD diagnosed between 1992 and 2007 in Shanghai Cancer Hospital were collected and analyzed for the incidence of prostate cancer. The median follow-up was 78 months. In total, 38 cases of invasive and 10 cases of *in situ* EMPD had been registered. A second malignancy was found in 28.9% (11/38) of patients with invasive EMPD and in 30% (3/10) of patients with *in situ* EMPD. Patients had an increased risk of developing a second cancer compared with the general population (standardized incidence ratio: 1.7; 95% confidence interval 1.2–2.4). Sixteen patients had serum prostate-specific antigen (PSA) level above 4 ng/mL; five developed prostate cancer, three of them with PSA levels beyond 100 ng/mL. The incidence of prostate cancer is 10.4% in this patient group. Patients with EMPD were more likely to have prostate cancer than the general population. Although the prognosis of EMPD is fairly good, a thorough search for a second tumor is recommended.

Paget disease (PD) was first described by Sir James Paget in 1874 (1), referring to a malignant entity involving the nipple of the breast. He also found that this clinical entity could involve other organs. In 1889, Crocker described lesions on the scrotum and penis that exhibited similar histologic features to mammary PD (mPD) and defined as extramammary Paget disease (EMPD) (2). Although it is a rare disease, EMPD is a marker for defects in the body’s cancer surveillance mechanisms and early detection of a second tumor could be lifesaving (3). However, there are little epidemiology data about the incidence of EMPD and its relationship to a visceral carcinoma. Moreover, no clear guidelines have been established for the diagnosis, treatment and follow-up of patients with EMPD.

In our clinical practice, we have found increased prostate-specific antigen (PSA) levels in patients with EMPD, leading to the investigation of prostate cancer. The aim of this population-based study was to evaluate the incidence of prostate cancer in patients with EMPD.

## Patients and methods

All cases of EMPD diagnosed from 1992 to 2007 were included in the study. The follow-up of EMPD was completed by December 1, 2009. All instances of EMPD in this study were histologically confirmed.

A total of 48 male patients with EMPD were registered at the Shanghai Cancer Hospital. A thorough physical examination and diagnostic tests, including serum tumor markers, cystoscopy, colonoscopy and chest/abdominal/pelvic computed tomography (CT) scan were used to exclude the presence of an internal noncutaneous malignancy. As EMPD may present as adenocarcinoma of a skin appendage, pathologic reviews were also performed in those patients.

The time between the diagnosis of EMPD and the second tumor, adjusted for age and calendar year of the EMPD diagnosis, was taken into account. The incidence of the second tumor (observed) was compared with the incidence of the same primary cancer in the general population (expected). The 95% confidence intervals were calculated using the Poisson probability. Values of p < 0.05 were considered statistically significant. Serum total PSA was measured by the method of time-resolved fluoroimmunoassay.

## Results

### Epidemiology

The mean age at diagnosis was 68 years (range 36–79 years, n = 48). Thirty-eight cancers were localized in the penoscrotal region; two on the extragenital skin, including one on the skin of the trunk and one in the groin; three on the axilla; and five in the perianal area (Table 1).

**Table 1:** Clinicopathologic features of the patients with EMPD

	*In situ* EMPD	Invasive EMPD	p-value
No. of patients	10	38	
Age (years)	71 (48–79)	61 (36–69)	<0.001
Longest diameter of the skin lesion (cm)	6 (1–20)	6.5 (5–18)	0.67
Percentage of metastatic EMPD	20	60	<0.001
Prostate cancer	1	4	0.23
Time to prostate cancer (years)	10 (5–15)	7 (5–13)	0.33

Data are presented as median (range). EMPD, extramammary Paget’s disease.

A second malignancy was found in 28.9% (11/38) of patients with invasive EMPD and 30% (3/10) of patients with *in situ* EMPD. Patients had an increased risk of developing a second cancer compared with the general population (standardized incidence ratio: 1.7; 95% confidence interval 1.2–2.4). The most frequent localizations of these cancers were the colorectum, the prostate, the breast and the extragenital skin (data not shown).

### Serum PSA level and incidence of prostate cancer in patients with EMPD

A total of 16 patients showed a PSA level above 4 ng/mL and this incidence of increase is 33.33% (16/48) vs 6.7% in the general population (4), among whom five had prostate cancer, as established by transrectal ultrasound biopsy (Table 2). The incidence of prostate cancer in this patient group is 10.4% (5/48), which is much higher than the incidence in the general population (4). The detailed clinicopathologic characteristics of the five patients are listed in Table 3.

**Table 3:** Clinicopathologic data of five EMPD patients with prostate cancer

Case	Age (years)^1^	Longest diameter of the skin lesion (cm)	Invasion	Serum PSA level (ng/mL)	CEA (μg/L)	Gleason score (biopsy)	Time to prostate cancer (years)	Outcome	Follow-up (months)
1	70	11	Subcutaneous tissue	166	52.4	3 + 3	6	RP	12
2	61	12	Subcutaneous tissue	101	1.2	3 + 3	11	RP	150
3	60	10	Subcutaneous tissue	89	20	3 + 4	3	ADT	60
4	65	7	Dermis	120	16	4 + 3	3	ADT	60
5	75	13	Subcutaneous tissue	38	22	4 + 3	8	ADT	120

EMPD, extramammary Paget’s disease; PSA, prostate-specific antigen; CEA, carcinoembryonic antigen; RP radical prostatectomy; ADT, androgen deprivation therapy.

1 At presentation with metastatic disease.

**Table 2:** Characteristics of PSA in 48 EMPD patients

PSA level (ng/mL)	Below 4	Above 4
Prostate cancer (N)	0	5
No prostate cancer (N)	32	11
Sum (N)	32	16

EMPD, extramammary Paget’s disease.

## Discussion

In this study, we found that EMPD patients had an increased risk of developing a second cancer and that patients with increased PSA levels had a higher incidence of prostate cancer. We therefore suggest a thorough search for a second tumor in EMPD patients.

The location of the underlying internal malignancy is linked to the location of the EMPD. Specifically, a perianal location may signify a malignancy of the gastrointestinal tract, while a penile, scrotal or groin location may be associated with an adenocarcinoma of the genitourinary tract. Chen et al. (5) found associated carcinomas in 31% of their patients while Pierie et al. (6) found concurrent secondary malignancies in 42% of their patients. Iwenofu et al. (7) found that the median age of their patients was 68 years, while Pierie et al. (6) found a median age of 70 years. Taking the patient age in the present study into account, patients with EMPD had 1.7 times higher risk of developing a second cancer during a follow-up period of 15 years. Chandra’s hypothesis that EMPD is a marker for deficits in the body’s cancer surveillance mechanism (3) could be supported by our study. About one-third of the tumor recurrences were adenocarcinomas, mostly originating from the colorectum, prostate and breast.

In this study, there are no concurrent primary malignancies or prior primary malignancies occurring during the follow-up period. Although we thoroughly searched for a recurrence of cancer after EMPD, new tumors could develop in the future. Therefore, the number of other cancers before and after EMPD could both be underestimated. The large number of other cancers in patients with *in situ* and invasive EMPD in this study supports the recommendation for a thorough search for other malignancies.

Overall, the prognosis for EMPD was fairly good. No treatment modalities or other tumors before or after EMPD tended to be associated with a higher risk of death. This finding was significant in univariate analyses, and thus without adjustment for age and sex. After adjusting for all variables, patients with no treatment had an elevated risk of death. Thus, the recommended diagnostic tests were a digital rectum examination (DRE), colonoscopy, CT of the abdomen and pelvis, and PSA testing in patients with penoscrotal EMPD. This increased risk may be caused by the fact that these patients had more comorbidities. The results of this survival analysis should be considered within the limitations of the small number of patients and probable underestimation of the number of other tumors.

In this study, five patients developed prostate cancer. If there is any communication between the prostatic duct system and the surface epidermis, the cancer could spread from the skin site to the scrotal skin. The likeliest route is via prostatic ducts traversing urethral epithelium. There is histologic proof that prostate cancer presenting as epidermal involvement originated from EMPD (8), just like Paget’s disease of the breast, the chemotaxis of these breast cancer cells, which eventually migrate into the overlying nipple epidermis (9). However, this theory remains unproven as there was no laboratory evidence of non-involvement of the scrotal area and nonlesional sites were not biopsied. It may be of interest to observe any change in surface skin involvement during follow-up of our patients. Another possibility is that PD of the nipple is a stem cell disease (10), meaning that EMPD may be a result of proliferation in the stem cell compartment.

Until now, Paget’s disease is a rare disease, and most of the reports are from single centers, especially in Asians. The disease may also be different in patients with a different ethnical background. In other words, it may be of interest to investigate the mechanism of EMPD through a multi-center cooperative study.

PSA positivity can be seen in cases of EMPD without associated adenocarcinoma of the prostate (11). The incidence of prostate cancer of EMPD patients is 10.4% (5/48) compared to 9.2% in the general population (4). Until now, no clear correlation between prostate cancer and EMPD has been demonstrated. There are also reports that immunohistochemical investigations of the tumor specimens from the prostate revealed an immunoprofile, which was very different from that of the primary skin lesion, and no case of EMPD with PSA positivity seems to represent an extension of an underlying prostatic adenocarcinoma (11). It is known that prostate cancer can be found in at least 1/3 of men in their 80s (12). The number of other tumors in this study was too small to determine the risk of developing a second tumor at a specific localization by means of a person-years analysis.

In 2004, the WHO noted that total serum PSA is still the best marker for the detection of prostate cancer, and the threshold PSA value for undergoing a biopsy was set at 4 ng/mL (13). Increased PSA levels were observed in 33% of the male EMPD patients[16/48] compared with 6.7% in the general population. We do not currently have an explanation for this finding.

The incidence of prostate cancer in EMPD patients whose serum total PSA was above 4.0 ng/mL is 31.25% (5/16). In the general population, for those with PSA levels above 4.0 ng/mL, prostate cancer is determined by biopsy in 25–30% of men evaluated (14). EMPD patient had a higher incidence of prostate cancer with PSA levels above 4.0 ng/mL, and interestingly, PSA levels are usually higher than 90 ng/mL in EMPD prostate cancer patients (80%, 4/5). However, further investigation is required.

Results from other studies have confirmed the prognostic value of the depth of skin invasion of EMPD (15). Our data did not show a difference in age between invasive and *in situ* EMPD, and prostate cancer incidence was also independent of EMPD type (invasive, *in situ*).

## Conclusions

The prognosis for patients with EMPD is fairly good, but a thorough examination for other tumors is recommended in addition to regular check-ups for at least 5 years, especially for patients with increased PSA levels. For EMPD on the male genital skin, the most likely sites of other tumors are the colorectum and prostate. Therefore, PSA and DRE are recommended for these patients.
